# Measuring anxiety-like behavior in a mouse model of mTBI: Assessment in standard and home cage assays

**DOI:** 10.3389/fnbeh.2023.1140724

**Published:** 2023-03-22

**Authors:** Liron Tseitlin, Bar Richmond-Hacham, Adi Vita, Shaul Schreiber, Chaim G. Pick, Lior Bikovski

**Affiliations:** ^1^Department of Anatomy and Anthropology, Tel Aviv University Sackler Faculty of Medicine, Tel Aviv, Israel; ^2^Department of Psychiatry, Tel Aviv Sourasky Medical Center, Tel Aviv, Israel; ^3^Dr. Miriam and Sheldon G. Adelson Clinic for Drug Abuse Treatment and Research, Tel Aviv Sourasky Medical Center, Tel Aviv, Israel; ^4^Sackler Faculty of Medicine and Sagol School of Neuroscience, Tel Aviv University, Tel Aviv, Israel; ^5^Sylvan Adams Sports Institute, Tel Aviv University, Tel Aviv, Israel; ^6^Dr. Miriam and Sheldon G. Adelson Chair and Center for the Biology of Addictive Diseases, Tel-Aviv University, Tel-Aviv, Israel; ^7^Myers Neuro-Behavioral Core Facility, Sackler Faculty of Medicine, Tel Aviv University, Tel Aviv, Israel; ^8^School of Behavioral Sciences, Netanya Academic College, Netanya, Israel

**Keywords:** TBI, anxiety, behavior, elevated plus maze, open field, marble burying test, PhenoTyper home cage, mental disorders

## Abstract

Traumatic brain injury (TBI) is a primary global health concern and one of the most common causes of neurological impairments in people under 50. Mild TBI (mTBI) accounts for the majority of TBI cases. Anxiety is the most common complaint after mTBI in humans. This study aims to evaluate behavioral tests designed to assess anxiety-like phenotypes in a mice model of mTBI. ICR mice underwent mTBI using the weight-drop model. Seven days post-injury, mice were subjected to one of five different behavioral tests: Elevated Plus Maze (EPM), Open Field apparatus (OF), Marble Burying test (MBT), Light Dark Box (LDB), and the Light Spot test within the PhenoTyper home cage (LS). In the EPM and OF tests, there were no significant differences between the groups. During the 30-min test period of the MBT, mTBI mice buried significantly more marbles than control mice. In the LDB, mTBI mice spent significantly less time on the far side of the arena than control mice. In addition, the time it took for mTBI mice to get to the far side of the arena was significantly longer compared to controls. Results of LS show significant within-group mean differences for total distance traveled for mTBI mice but not for the control. Furthermore, injured mice moved significantly more than control mice. According to the results, the anxiety traits exhibited by mTBI mice depend upon the time of exposure to the aversive stimulus, the apparatus, and the properties of the stressors used. Therefore, the characterization of anxiety-like behavior in mTBI mice is more complicated than was initially suggested. Based on our findings, we recommend incorporating a variety of stressors and test session lengths when assessing anxiety-like behavior in experimental models of mTBI.

## Introduction

Traumatic brain injury (TBI) is the most common neurological disorder among young adults and the elderly, with an estimated 10 million cases a year worldwide (Menon et al., 2010; Dewan et al., 2019). TBI is a disruption of normal brain function caused by an external force applied to the head or body (Menon et al., 2010). This injury can result from occupational or sports accidents, military injuries, motor vehicle collisions, and falls (McAllister, 2016; Taylor et al., 2017). Individuals who experience TBI may present a broad range of short and long-term cognitive, emotional, behavioral, sensorimotor, and functional deficits, which vary due to the injury’s type and severity (Silver et al., 2019). The pathophysiology of head injuries can be divided into primary and secondary damage. Initially, physical injury can cause damage to the blood vessels, brain tissue, and the blood-brain barrier (BBB). Subsequently, secondary brain damage (edema, inflammation, pro-inflammatory cytokines, free radicals, glutamate-induced toxicity, and DNA damage) gradually progresses over time (days to months). Secondary damage, if not treated, will eventually cause cell death (Reilly and Bullock, 2005; Griesbach et al., 2007; Rachmany et al., 2013).

TBI can be classified into three main categories: mild, moderate, or severe (Mallya et al., 2015), of which mild traumatic brain injury (mTBI) is the most common (Silverberg et al., 2020). American Congress of Rehabilitation Medicine (Kay et al., 1993) describes mTBI as a mild insult to the head that results in a brief period of unconsciousness followed by impaired cognitive function. Along with cognitive impairments, mTBI causes an array of symptoms, most notably headaches, fatigue, depression, anxiety, and irritability, collectively referred to as post-concussion syndrome (PCS; Kay et al., 1993). In most cases, the symptoms resolve within approximately 3 months. However, some individuals continue to experience symptoms beyond 1-year post-injury (Hall et al., 2005; Daneshvar et al., 2011). Those with persistent symptoms are said to experience persistent PCS (Daneshvar et al., 2011; Marshall, 2012). Moreover, mTBI is associated with a higher incidence of psychiatric disorders, up to 70% of which are anxiety disorders (Hibbard et al., 2000; Fann, 2004; Bryant, 2010).

Anxiety disorders are the most prevalent and earliest mental manifestations following TBI, ranked as the ninth most health-related cause of disability by the World Health Organization (WHO). Due to their enormous economic impact, anxiety disorders are a heavy burden on societies worldwide, with the highest prevalence in high-income countries (Bandelow and Michaelis, 2015; Stein et al., 2017; Vestergaard et al., 2020; Penninx et al., 2021). The Diagnostic and Statistical Manual of Mental Disorders (DSM-V) defines anxiety disorders as disorders that share features of excessive fear, anxiety, and related behavioral disturbances. For example, fear is the emotional response to a real or perceived imminent threat, whereas anxiety is the anticipation of future threats (American Psychiatric Association, 2013). There are several types of anxiety disorders, and they tend to be highly comorbid with each other. However, they can be differentiated by the conditions that induce fear, anxiety, or avoidance behavior and their clinical manifestations (American Psychiatric Association, 2013; Penninx et al., 2021). Following a TBI, anxiety disorders are classified as “due to a general medical condition” and may include panic disorder (with or without agoraphobia), general anxiety disorder, post-traumatic stress disorder (PTSD), and obsessive-compulsive disorder (OCD; Moore et al., 2006). However, nowadays (in DSM-V), OCD and PTSD are no longer classified as anxiety disorders (American Psychiatric Association, 2013).

A variety of behavioral tests are used in anxiety-like condition research conducted in pre-clinical settings. These tests assess, in mice, the conflict between exploring novel areas and the fear of open spaces and bright light. These include the Open Field Test, the Elevated Plus Maze, the Elevated Zero Maze, the Light Dark Box, the Marble Burying test, the Hole-Board test as well as Automated Home Cage Observations (Visser et al., 2005; Moore et al., 2006; Himanshu et al., 2020). However, anxiety-like behavior has been challenging to reproduce in animal models. Further, the literature offers conflicting findings regarding the effect of a TBI on anxiety, with some studies reporting an increase, others a decrease, or no change at all (Popovitz et al., 2019; Tucker and McCabe, 2021).

While mTBI plays a notable role in the occurrence of anxiety in humans, anxiety-like behavior is often elusive in pre-clinical studies. Therefore, the current study aims to assess various behavioral tests, thus shedding light on the advantages and disadvantages of each one of them.

## Methods

### Mice and experimental design

Adult male ICR mice (HSD, Israel), 6–8 weeks old, weighing 30–40 grams, were used once in this study. Mice were housed in groups of five in a cage, in 12-h light/dark circles, at a constant room temperature of 22°C, and allowed *ad libitum* access to food and water. After arriving at the facility, the animals were given seven days to adjust to their new environment. The minimum number of mice was used to facilitate results, and all effort was made to ease the mice’s suffering throughout the experiments. Each group of mice was randomly assigned to one of the experimental groups. To minimize anxiety and to ensure consistency in post-mTBI time, each group of mice underwent a single behavioral test All behavioral tests were conducted under red light conditions during the active phase. The study was conducted in the Myers Neuro-Behavioral Core Facility at the Sackler Faculty of Medicine, Tel Aviv University. In accordance with the NIH guidelines for animal care, all procedures were approved by the ethics committee of the Sackler Faculty of Medicine (01-21-069) (DHEW publication 85-23, revised, 1995).

### Mild traumatic brain injury procedure

A mild traumatic brain injury was induced using a weight-drop model (Zohar et al., 2003). The weight drop concussive head injury device comprises an aluminum tube with an internal diameter of 13 mm and a length of 80 cm. Each mouse was anesthetized with isoflurane to induce injury and placed under the device on top of a sponge. Next, we released a 30-g weight at the beginning of the tube, free-falling along its length. This weight struck the right lobe of the mouse’s brain between its eye and ear. Once the procedure had been completed, the mice were returned to their home cages for recovery and follow-up. To ensure that any deficits observed are only related to the mTBI, the control mice were also anesthetized with isoflurane.

### Behavioral assessment

Mouse exploration and anxiety-like behavior were assessed using the Elevated Plus Maze (EPM), Open Field apparatus (OF), Marble Burying test (MBT), Light Dark Box (LDB), and the Light Spot (LS) test within the PhenoTyper home cage (model 3000, Noldus Information Technology, The Netherlands). Behavioral tests were conducted in a counterbalanced order in separate groups of mice seven days after injury. A total of 108 mice (mTBI and control) were placed individually in the arena of each test, and behavior was recorded. Data was collected with Ethovision 15.0 software (Noldus Info Tech, Wageningen, The Netherlands; Noldus et al., 2001). All behavioral tests were performed at the beginning of the active cycle (dark phase) and under red light. A total of 108 mice (mTBI and control) were placed individually in the arena of each test, and behavior was recorded. Data was collected with Ethovision 15.0 software (Noldus Info Tech, Wageningen, The Netherlands; Noldus et al., 2001). All equipment was cleaned with a 5% Virusolve solution between subjects to eliminate residual odors.

#### Elevated plus maze (EPM)

EPM relies on the rodent’s dual (contrasting) tendencies toward novel places, with both a desire to explore and to avoid unfamiliar open spaces (Lister, 1987; Har-Even et al., 2021). The apparatus consists of a four-armed black plexiglass platform formed into a “plus” shape suspended 50 cm above the ground. The arms are confined by either low (“open”; 30 × 5 × 1 cm) or high (“closed”; 30 × 5 × 15 cm) walls, such that similarly shaped arms face one another. All tests were done with an array of four red light (dark conditions) lamps located above the maze with a maximum of 50 LUX intensity in the open arms. Mice were placed individually at the central platform, facing an open arm. The mice were allowed to move freely for 5 min. Time spent in the open arm and the entries to the open and closed arms were measured.

#### Open field (OF)

The OF test provides a way to assess novel environment exploration and general locomotor activity systematically (Hall, 1934; Walsh and Cummins, 1976) In addition, it provides an initial screen for anxiety-like behavior in rodents (Namdar et al., 2020). Each mouse was placed individually in the center of an open-field plexiglass box (60 × 60 × 20 cm), and its behavior was recorded for 5 min. The total distance traveled and the cumulative duration of the time the mouse remained in the center of the arena were measured to assess anxiety-like behavior.

#### Marble burying (MBT)

The MBT is used to measure anxiety-like behavior in correlation with the number of marbles buried (Chaudhary, 2016). Standard polycarbonate cages (22 × 30 × 28 cm) were used, and odorless bedding material was placed inside the cage, 5 cm in depth. Twenty marbles were evenly distributed across the bedding. Then, the mice were separately put in the marble cage for 30 min. At 30 min all marbles that were unburied were counted (less than 2/3rd of the marble’s height).

#### Light dark box (LDB)

This test is based on animals’ natural conflict between seeking protection and exploring a novel environment (Shanazz et al., 2021). The apparatus consisted of two chambers. One is an open-field plexiglass box (60 × 60 × 20 cm), and the second is a dark box (16 × 35 × 16 cm). The light chamber was illuminated with 822-lux light. Each mouse was individually placed in the dark box and allowed to freely explore the apparatus for 10 min. The total distance traveled, the time spent on the far side of the chamber, and the latency to reach the far side of the chamber were recorded.

#### The light spot (LS) test within the PhenoTyper home cage

Mice were housed individually in PhenoTyper home cages for four days. The first three days were considered habituation days in which mice were allowed to familiarize with the new home cage environment. During these 4 days, no human handling took place; food and water were provided ad libitum. The cages (30 × 30 × 30 cm) are made of transparent plexiglass walls with an opaque plexiglass floor covered with bedding. The cages are equipped with a feeding station, a water bottle, a running wheel, and a shelter. The cage’s lid is equipped with infrared LEDs and an infrared-sensitive camera for video tracking. EthoVision was used as video tracking and trial control software (EthoVision XT 14, Noldus Information Technology, The Netherlands; Grieco et al., 2021). On the day of the LS test, 3 h after the beginning of the dark (active) phase, a bright LED light (1,100 LUX) was automatically switched on for 1 h, illuminating the feeding station. To examine the behavioral response to the LS, we measured the total distance traveled (in centimeters) by mice before the LS was turned on (00:00–02:00 h, system time), during the LS test (03:00 h, system time), and after the LS was switched off (04:00–08:00 h, system time; Maluach et al., 2017; Prevot et al., 2019).

### Statistical analysis

The statistical analysis for the various tests was performed using SPSS software and RStudio. For the non-automated test data, t-tests were used to examine potential differences between the groups. LS test data were analyzed with a Linear Mixed Effects Regression (LMER) model using the function lmer from the R packages “lmer4” and “lmerTest” (Bates et al., 2015). The model included group and LS phase as fixed effect terms and the animal identifier (ID) as random effect terms to account for repeated measurement. Significant group × LS phase interaction effects were followed up with Sidak’s multiple comparisons using the “emmeans” function from the “emmeans” R package (Chaudhary, 2016; Lenth et al., 2019). All tests were two-tailed, and *p* < 0.05 was considered statistically significant. Statistically significant differences were marked with asterisks; *p < 0.05, **p < 0.01, ***p < 0.001.

## Results

All the groups were assessed for anxiety-like behavior seven days post mTBI *via* five different tests. All tests are based on the conflict between rodents’ tendency to explore a novel environment and the fear of open and/or brightly lit spaces.

### Elevated plus maze

**Figure 1:**
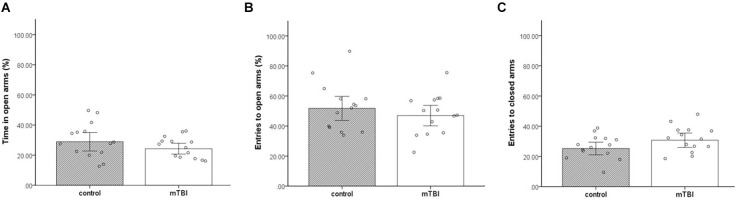
Elevated plus maze. **(A)** Mean ± SEM of the time spent in the open arms of the elevated plus maze in %. **(B)** Mean ± SEM of the entries to the open arms in %. **(C)** Mean ± SEM of the entries to close arms (*n* = 14 in each group).

No significant differences were found between the groups in all three parameters; time in open arms, entries to open arms, and entries to close arms (*t*_(1,26)_ = 1.3, *p* = 0.21, *t*_(1,26)_ = 0.7, *p* = 0.5, *t*_(1,26)_ = <1.7, *p* = 0.09, respectively) in anxiety-like behavior and general locomotor activity examined in the EPM. Mice exposed to mTBI spent a similar amount of time in the EPM open arms as the control mice (24.3 ± 6.7 and 30 ± 11.5, respectively; Figure 1A). In addition, mTBI mice entered open}close arms in a similar manner (47.8 ± 12.2 and 51.6 ± 15.4, 30.7 ± 9 and 25.3 ± 8 respectively; Figures 1B,C).

### Open field

**Figure 2:**
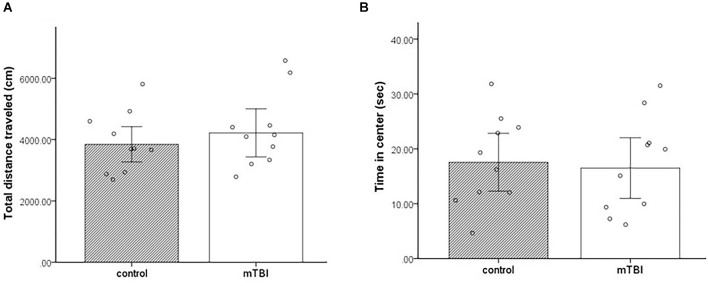
Open field. **(A)** Total distance traveled as mean ± SEM in cm. **(B)** Time in the center as mean ± SEM of cumulative duration in seconds (sec) (*n* = 10 in each group).

Two variables were taken from the open field paradigm—the total distance traveled and the total time spent in the center of the arena. In both parameters, there were no significant differences between the groups *t*_(1,18)_ = −0.75, *p* = 0.46, *t*_(1,18)_ = 0.3, and *p* = 0.8, respectively. mTBI mice presented similar general locomotor activity as control mice (distance 4,219.6 ± 1,264 and 3,847.3 ± 930.8, respectively). In addition, both mTBI and control mice spent comparable amounts of time in the center of the arena (16.5 ± 9 and 17.6 ± 8.5, respectively; Figure 2).

### Marble burying

**Figure 3:**
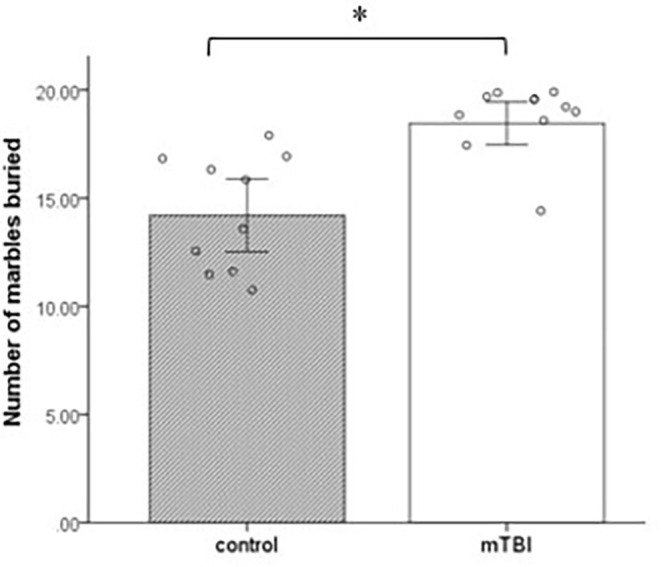
Marble burying. Mean ± SEM of the marbles buried by the mice after 30 min (*n* = 10 in each group). *p < 0.05.

Marbles buried with bedding up to 2/3 of their depth were counted at 30 min. The groups differ significantly in the number of marbles buried during the test period. mTBI mice buried a significantly greater number of marbles than control mice at a 30-min test period (*t*_(1,18)_ = 4.2, *p* = 0.001; Figure 3).

### Light dark box

**Figure 4:**
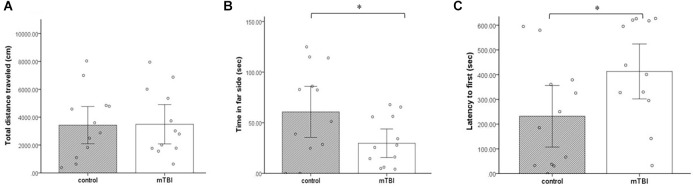
Light dark box. **(A)** Total distance traveled as mean ± SEM in cm. **(B)** Far side as mean ± SEM of cumulative duration in the far side of the arena in seconds (sec). **(C)** Latency to first as mean ± SEM of cumulative duration of the time past until the first time in the far side of the arena in seconds (*n* = 12 in each group). *p < 0.05.

Multiple measures were taken from the LDB test. No significant differences between the groups were observed in the total distance (*t*_(1,22)_ = −0.06, *p* = 0.9; Figure 4). However, in contrast to the lack of difference between the groups in cumulative duration of time in the center of the arena (OF), here, the cumulative duration of control mice on the far side of the arena was significantly higher than the time the injured mice spent there (*t*_(1,22)_ = −2.1, *p* = 0.047; Figure 4). In addition, latency to first (the time it took for mice to get to the far side of the arena) was significantly longer in the mTBI group compared with the control group (*t*_(1,22)_ = 2.1, *p* = 0.045; Figure 4).

### Light spot (LS) test in the PhenoTyper home cage

**Figure 5:**
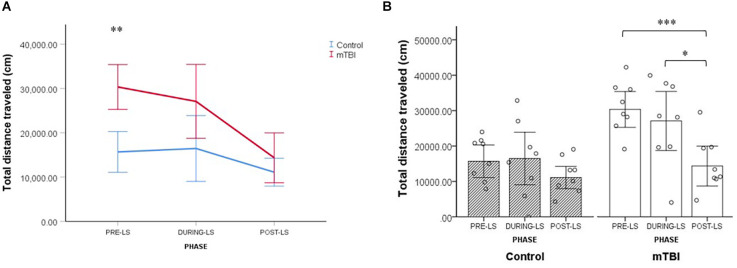
LS test (Light Spot test). **(A)** Group by LS phase interaction effect on the total distance traveled by mTBI (*n* = 8) and control mice (*n* = 8). Values are mean ± SEM. **(B)** Within-group comparisons for control and mTBI mice across the different LS test phases. Values are mean ± SE. *p < 0.05, **p < 0.01, ***p < 0.001.

Results of a Linear Mixed Model analysis revealed that injury had an effect on total distance traveled, with mTBI mice ambulating overall greater distances than control mice (main effect of group: *F*_(1,18.86)_ = 8.10, *p* = 0.010). Furthermore, the distance traveled by the two groups differed across test phases (group × test phase interaction: *F*_(2,108)_ = 4.11, *p* = 0.019; Figure 5A).

In order to further explore this interplay between the experimental group and the test phase, we conducted a series of follow-up analyses. First, we compared the total distance traveled by mTBI mice and control mice in each of the three test phases. It was found that the distance traveled by mTBI mice differed significantly from that of controls only in the pre-LS stage (mTBI mice: 30,350.3 ± 8,754.7, control mice: 15,684.6 ± 9,324.4; *p* < 0.001; Figure 5A).

Next, we compared the total distance traveled in the three test phases separately for the mTBI and control groups. Results indicate that only in mTBI mice did LS exposure result in a significant reduction of total distance traveled. Among injured mice, the distance ambulated after LS exposure was significantly shorter than before the exposure (14,356.7 ± 13,070.3 vs. 30,350.3 ± 8,754.7; *p* < 0.001) and during the exposure (14,356.7 ± 13,070.3 vs. 27,092.1 ± 11,796.4; *p* = 0.004; see Figure 5B). The distance traveled by control mice did not differ significantly over the three test phases (all *p* > 0.05; Figure 5B). Collectively, the data from the LS test in the home cage indicate the presence of more persistent anxiety-like behavior in the mTBI group, that is, residual avoidance behavior (Kyriakou et al., 2018; Grieco et al., 2021; Figures 5A,B).

In summary, EPM and OF were each 5 min long with a new and open arena as a stressor. In both tests, there were no significant differences between the groups. The LDB was a 10 min evaluation with a bright light stressor added to the new and open arena. Like in the OF, there were no significant differences between the groups in total distance traveled. Nevertheless, the cumulative time spent on the far side of the arena was significantly lower for mTBI mice than for control mice. In addition, latency to first was significantly longer in mTBI mice compared to controls. The MBT was a 30-min evaluation with a stressor of 20 shiny marbles. During the 30-min test period, mTBI mice buried a significantly increased number of marbles than the control mice. The LS test was unique, conducted over hours and integrating two stressors: social isolation and bright light. Significant within-group mean differences for total distance traveled were found for the mTBI group but not for the control group. In addition, the total distance moved by injured mice (in all test phases together) was significantly higher than the control group.

## Discussion

In previous studies done in our lab using the same mTBI protocol, we established that mTBI affects learning and memory as well as gait and balance, and sleeping patterns in mice (Rubovitch et al., 2019; Namdar et al., 2020; Richmond-Hacham et al., 2022). The current work aims to elaborate on anxiety. This study aimed to evaluate anxiety-like behavior in an mTBI mouse model, using four different gold standard assays and one home cage environment. Each behavioral test was performed on different groups of mice. The study design was chosen to keep the stressor of one test from affecting anxiety levels in the following test. In addition, it is well known that the time point post-TBI is an extremely relevant parameter when studying anxiety (Popovitz et al., 2019). Nevertheless, the design may affect the ability to compare and speak about the tests as a unit. We assessed anxiety tests that fall into three categories: Tests to evaluate open space-induced anxiety (EPM and OF), tests of novelty-induced anxiety (MBT), and tests of light exposure-induced anxiety (LDB and LS). Only male mice were used in this study since head injuries are significantly more common in men (Cassidy et al., 2004). In addition, it has been shown that the estrogen secretion circuit affects anxiety levels (Walf and Frye, 2006). Indeed, conducting a similar study in female mice would be a desirable next step. Our findings reveal that the detection of anxiety-like behavior in a mice model of mTBI depends on both the test employed (related stressor) and the exposure duration.

Among the more “traditional” anxiety-like tests, the results suggest that the EPM and OF, open space-based anxiety tests, are the least sensitive paradigms, in line with existing literature (Ennaceur and Chazot, 2016). These findings may have changed if a different TBI model or severity had been used. For example, as reported in Popovitz et al. (2019) article from 2019, moderate to severe CCI models increased anxiety measured by EPM up to three weeks post-injury. In contrast, moderate to severe TBI in our weight drop model did not show an increase in anxiety by EPM (Heim et al., 2017; Har-Even et al., 2021; Qubty et al., 2022). Moreover, despite no differences between the two groups in the time spent within the open arms of the EPM and the center of the OF arena, mTBI mice spent significantly more time in the outer area of the LDB arena than control mice. The exposure duration to the stressor of the LDB test is longer compared to the EPM and OF paradigms. However, when considering evidence showing that there are no significant differences between mTBI and control mice in OF when exposures last longer (Namdar et al., 2020), we suggest that the difference between the tests is primarily a consequence of the light exposure added to the new and open arena. Moreover, based on evidence from non-TBI research (Ennaceur et al., 2006), it may be that the sensitivity of inner arena activity parameters (OF) is not the same as that of outer arena activity parameters (LDB). The 30-min long MBT test of novelty-induced anxiety further supports our assumption regarding the vital role of exposure duration. It was found that mTBI mice buried more marbles at 30-min test period, indicating increased anxiety levels. Therefore, a short test session might have allowed this result to be overlooked, leading to an erroneous conclusion that mTBI does not alter novelty-induced anxiety responses. Moreover, we can conclude from this finding that the “count of marbles buried” may be particularly susceptible to the effect of exposure duration.

A further indication of the importance of exposure duration is the observation that only the automated LS test, the test with the most extended duration, was sensitive to anxiety-induced changes in total distance traveled among mTBI mice. Notably, when measured as part of the OF or LDB tests, the same parameter failed to indicate differences between the two groups. This result illustrates the influence of not only stressor exposure duration but also the behavioral test or anxiety domain being evaluated as well as the interplay between them. While all four standard assays are performed in a novel arena, the LS is conducted in a home cage environment. It is presumed that mice respond differently when the aversive stimulus is in their home. More concretely, open space-based paradigms (OF, EPM), novelty-based paradigms (marble burying), and light-based paradigms (LDB, LS) are known to measure different aspects of the multidimensional and complex nature of anxiety (Kalueff et al., 2007). Consequently, impairments in certain anxiety subdomains, like avoidance, may take longer to manifest and thereby require a longer time to be detected (Kyriakou et al., 2018). The anxiety-like behavior of mTBI mice in the LS test supports this assertion. It emphasizes the importance of studying subsequent time points following aversive stimuli (even up to five hours later) rather than focusing primarily on the acute response (Kyriakou et al., 2018). It may be especially true for low anxiety strains of mice, such as those in this study (Ennaceur, 2014), that longer exposure duration may be needed to elicit anxiety response when mild aversive stimuli are used. An additional consequence of prolonged behavioral tests could be fatigue. It is observed that mice, like humans, alter their sleep patterns following a traumatic brain injury (Schreiber et al., 2008; Namdar et al., 2020). Given that sleep disruption has been shown to exacerbate emotional vulnerability and increase anxiety (Chellappa and Aeschbach, 2022), it is most likely that its contribution to mice’s exhibition of anxiety-like behavior will emerge in longer-duration tests.

The methods chosen here enabled us to account for the intensity of the aversive stimuli (i.e., only mild stressors were employed), expanding empirical knowledge and clearing some of the conceptual fog surrounding the relationship between mTBI and anxiety. In that aspect, it may resemble, as much as possible, “real life” situations (relatively mild stressors) that mTBI patients may encounter daily and react differently than non-mTBI patients. Clinically thinking, numerous brain regions are associated with anxiety, such as the amygdala, hippocampus, anterior cingulate cortex, and many more (Engels et al., 2007; Litvin et al., 2008). Considering that brain injuries in humans are heterogenic in mechanism and severity, it is likely that they will result in different types of anxiety. Additionally, as we showed in a mice model, post-injury anxiety can be context-dependent in humans, meaning that the severity and presentation of anxiety symptoms may vary based on the circumstances and environment in which the individual finds themselves. For example, someone with TBI-related anxiety may feel more anxious in crowded or noisy environments or when faced with certain stressors that are reminiscent of the original injury (Mallya et al., 2015; Howlett et al., 2022). In pre-clinical settings, several types of TBI models are available: Fluid Percussion Injury Model (FPI), Controlled Cortical Impact Injury Model (CCI), Weight-Drop TBI Model, Penetrating Ballistic-Like Brain Injury Model, Blast Injury Model, Repetitive Brain Injury Model (Xiong et al., 2013). Compared to other TBI models, the weight-drop model used in the current study better mimics the injury processes in most human cases (McNamara et al., 2020). However, when conducting a study in a lab environment according to standardization guidelines, we cannot expect the heterogeneity we see in humans.

In this context, another factor to consider regarding the generalizability of our findings is the use of the ICR mouse strain. The ICR strain is one of the preclinical research’s most widely used outbred mice. The use of ICR mouse strain, on the one hand, elicit results that bear a resemblance to the heterogeneity in human and, on the other, are very strain specific. ICR mice have distinct characteristics that may affect outcomes of behavioral tests, such as a high level of general locomotor activity and resiliency to aversive stimuli (Adams et al., 2002; Ennaceur, 2014). This aspect is particularly relevant in light of the fact that the LS test is conducted in single-housing conditions. Accordingly, isolation-induced anxiety may have an additive effect, even though evidence from non-TBI research suggests that this is unlikely (Kamakura et al., 2016). It is, therefore, necessary to conduct further studies on mTBI in other mouse strains to clarify the role of injury in anxiety. Moreover, it is to be expected that there will be an environmental effect on the anxiety levels of mice in group housing. It is important to take into account the anxiety induced by returning mice to cages after being exposed to an aversive stimulus. However, in a non-TBI study, researchers showed that within-cage order of testing does not interfere with anxiety levels tested as the amount of plasma corticosterone (Benedetti et al., 2012). In addition, we see different results in tests done in group-housed mice. Consequently, if there is an effect it is not significant.

In summary, our results show that mTBI mice exhibit anxiety-like behavior in the MBT, the LDB, and the light-spot test but not in the EPM and OF tests. The relevance of testing several behavioral paradigms when evaluating anxiety-like behavior is highlighted. Also emphasized is the importance of increasing measurement times and using advanced automated tests in combination with the standard traditional tests.

## Data availability statement

The raw data supporting the conclusions of this article will be made available by the authors, without undue reservation.

## Ethics statement

The animal study was reviewed and approved by The ethics committee of the Sackler Faculty of Medicine.

## Author contributions

CP and LB contributed to conception and design of the study. LT and AV performed the experiments. BR-H performed the statistical analysis. LT wrote the manuscript. BR-H and AV wrote sections of the manuscript. CP and SS edited the manuscript. All authors contributed to the article and approved the submitted version.
